# The Real King Edward the VIIth: Lord Redesdale's Experiences
**Memories.* By Lord Redesdale, G.C.V.O., K.C.B. London: Hutchinson and Co.


**Published:** 1915-12-04

**Authors:** 


### THE REAL KING EDWARD THE VIITH.

### Lord Redesdale's Experiences.*

To all who are interested in the study of individual character, especially where the object to be studied is a great man or woman, no testimony is worthier of accord than that of the intimate friends of the person whose character is under review. The historian or outside observer is bound merely to present an oblique view of the essential traits which contributed most largely to the biography which he records; the personal friend, despite the probable indulgence which one may legitimately expect, is bound, at first-hand, to bring into prominence the intimate features which guided and maintained the character which he describes. Perhaps in no recent instance has the discord between the would-be historian and the personal friend been more sharply defined than in Lord Redesdale's "Memories," so far as they relate to the character and personality of King Edward VII. Sir Sidney Lee, with no intimate knowledge of the King, is responsible for the appreciation of him in the "Dictionary of National Biography"—and this is how Lord Redesdale views the situation:—

"'The Dictionary of National Biography' . . . will remain on the shelves of every library, public and private, for many generations, and will be consulted as an authority long after the writers, like their subjects, shall have faded into the misty land of ghosts. That is why articles in such an important book of reference should be subjected before publication to the strictest and most impartial examination. Afterwards it is no use. 'The written word stands.' Even should Sir Sidney Lee himself, in the fuller life of King Edward upon which he is said to be engaged, endeavour to modify, soften, or even contradict some of the statements in his article, it will not be possible for him to correct the false impression which those pages will create in the minds of men of a future generation. Historians will turn to them and will say that since this was written immediately after the tragedy of 1910 by so eminent a man of letters it must represent the contemporary judgment of the King's personality. Great is the responsibility."

### A Notable Personality.

To our view Lord Redesdale forms far too severe an opinion of the possibility of future judgment, being misled by this or other writing from the same source. King Edward's history and character are not likely to suffer from the "National Biography" article any more than that article is likely to escape from the dignified chastisement with which it has been visited by Lord Redesdale. He holds that the reader of the future will rise from the study of the article in the "National Biography" with an altogether false appreciation of its subject. He would see in it the portrait of a man with many lovable characteristics indeed, but with little conception of the high functions to which he was called; he would see a Prince self-indulgent, impatient of duty, with little political acumen even in those matters of foreign policy in which he took the highest interest; giving little concern to home affairs, "unremitting in his devotion to social pleasures"; showing "aloofness from the working of politics and a certain disinclination hastily to adapt his private plans to political emergencies." It is from this view that Lord Redesdale strongly dissents; to him, as to all those of his subjects who were privileged to be brought in contact with King Edward, his tact, "his magically conciliatory charm, a power of fascination which can rarely have been equalled, his judgment of men, have been universally acknowledged. He carried into public affairs a sympathy and kindliness which bore rich fruit. He could feel with a Gambetta as he could feel with the proud chieftain of the Hapsburgs. To a Scottish manse, to a Norfolk parsonage, he could carry the sympathy of a friend, the true message of love. He could enter into the troubles of a humble cottager on his estate with as much interest as he could listen to the family difficulties of a Duke. Above all, he could forgive, and that is perhaps the rarest of human powers. Those who know could cite more than one instance of its exercise. Nor was all this confined to mere words. He would spend himself on behalf of a friend. . . . His courage was beyond proof."

"My recollection of the King which I wish to place on record is that of a character made up of various qualities—a monarch deeply impressed with the duties and obligations of his exalted station; a man intensely human, and, let his critics say what they will, altogether lovable." Extended personal knowledge and intimate association as fellow-workers enable us to confirm the supreme justice and truth of Lord Redesdale's testimony.

### Educative Disabilities.

It is not everyone who is aware of the very dull and rigid restrictions under which the education of Prince Edward was conducted. Naturally full of good spirits and boisterous health, he was deprived of all school games and free companionship with boys of his own age. Even at Oxford it would appear that he took no part in the ordinary undergraduate life. One can therefore well imagine how much he enjoyed commencing his soldiering with the Grenadier Guards at the Curragh. It is interesting to learn that the strict régime under which the Prince was educated precluded all practical knowledge of common humanities and rendered it impossible for him to glean for himself by wide reading of the best books of the day the knowledge of men and matters which most boys are only too delighted to acquire. The gentlemen appointed to attend on him were directed that some of his leisure time was to be devoted to "music, to the fine arts, either drawing or looking over drawings, engravings,

* *Memories.* By Lord Redesdale, G.C.V.O., K.C.B. London: Hutchinson and Co.

etc., to hearing poetry, amusing books or good books read aloud!" No mention is made apparently of any leisure time being spent in a personal study of well-known books or of literature generally. In present days this sounds a poor equipment for any boy who is intended to take hold of, direct, and control his ordinary walk through life; for the heir-apparent of this Empire it sounds Gilbertian. And yet, no doubt in complete oblivion of the Prince's early training, critics are to be found who call attention to his reported distaste for literature. How, then, was it that, despite his early training, the Prince always impressed those with whom he dealt with his remarkable practical knowledge? According to Lord Redesdale, this was largely acquired through conversation and the Press. "His memory was phenomenal: he seemed unable to forget. The business of kingcraft is not one that is easy to learn. It is impossible for a king to specialise in any one subject, but he must be sufficiently posted in the trades of all sorts and conditions of men to be able to discuss intelligently the subjects upon which they have to address him. This King Edward did to perfection, and we must remember that this power was not acquired all of a sudden, like a miracle conferred upon him by anointment at his Coronation; it was the result of long years of patient listening and inquiry—of those same long years which his detractors would have us believe were spent to exhaustion in the pursuit of frivolous occupations, and in the selfish sacrifice of duty to pleasure. No more false charge was ever brought against a man in his exalted position." As an instance of the ignorance of such an imputation the following extract will suffice:

"One night I was dining at the Club, after King Edward had come to the throne, but before he had moved from Marlborough House into Buckingham Palace. He knew that I was in London for two or three days alone, so he sent over to ask whether I was at the Club, and if so to bid me go across to him. I found him in his private sitting-room, all alone, and we sat smoking and talking over old times for a couple of hours. Towards midnight he got up and said: 'Now I must bid you good-night, for I must set to work'—pointing to a huge pile of the familiar red boxes. 'Surely,' I said, 'your Majesty is not going to tackle all that work to-night!' His answer was: 'Yes, I must! Besides, it is all so interesting'; and then he gave me one of his happy smiles, and I left him. 'So interesting!'—that was the frame of mind in which he faced his work—he the man who we are expected to believe could not be brought to attend to business!"

### King Edward and the Hospitals.

To no one who has in the slightest way concerned himself with medical institutional life can King Edward be regarded as otherwise than the greatest instigator of reform and progress. Let one reflect for a few moments upon the deep interest he took in all that concerned the sick and suffering; let one go into the past history and recorded results of the recreation of Guy's Hospital, King Edward VII.'s Hospital Fund, the League of Mercy, and the Royal National Pension Fund for Nurses—taking four notable instances of his interest; and, leaving out of account all the other benefits that he bestowed, can any reader say that through the whole course of our history greater good has been done in any other reign? He was no mere figurehead conferring his patronage and leaving subsequent direction entirely in the hands of local administrators. Knowledge and the best way to administer and control were the surest steps on the road towards his personal interest and favour. Last and greatest work of all, we see to-day that his interest in foreign affairs has placed this Empire most closely in touch with the European Powers who are welded together with the firm resolve to crush and dissipate the German attempt to destroy liberty, freedom, and the well-being of the people everywhere, with all we hold to be best in the civilisation of the world.

